# Exploring pre‐hospital healthcare workers' readiness for chemical, biological, radiological, and nuclear threats in the State of Qatar: A cross‐sectional study

**DOI:** 10.1002/hsr2.803

**Published:** 2022-08-30

**Authors:** Hassan Farhat, Guillaume Alinier, Padarath Gangaram, Kawther El Aifa, Mohamed Chaker Khenissi, Sonia Bounouh, Moncef Khadhraoui, Imed Gargouri, James Laughton

**Affiliations:** ^1^ Hamad Medical Corporation Ambulance Service Doha Qatar; ^2^ Faculty of Sciences University of Sfax Sfax Tunisia; ^3^ Faculty of Medicine “Ibn El Jazzar” University of Sousse Sousse Tunisia; ^4^ School of Health and Social Work University of Hertfordshire Hatfield UK; ^5^ Weill Cornell Medicine College Doha Qatar; ^6^ Faculty of Health and Life Sciences Northumbria University Newcastle upon Tyne UK; ^7^ Faculty of Health Sciences Durban University of Technology Durban South Africa; ^8^ Departement of Analytical Chemistry and Environmental Pollution, Higher Institute of Biotechnology University of Sfax Sfax Tunisia; ^9^ Faculty of Medicine University of Sfax Sfax Tunisia

**Keywords:** HazMat‐CBRN, reliability, satisfaction, training, validity

## Abstract

**Background:**

Hazardous Material—Chemical, Biological, Radiological, and Nuclear (HazMat‐CBRN) incidents, though infrequent, are environmentally precarious and perilous to living beings. They can be deliberate or accidental or follow the re‐emergence of highly contagious diseases. Successful management of such incidents in pre‐hospital settings requires having well‐trained and prepared healthcare workers.

**Aims:**

This study aimed to explore the reliability and validity of a satisfaction survey, answered by Specialized Emergency Management (SEM) personnel from a national Middle Eastern ambulance service, with a “Hazardous Material Incident Management” course offered to them as a continuing professional development activity and seek their opinion regarding Hamad Medical Corporation Ambulance Service personnel needs for other HazMat‐CBRN related training topics.

**Method:**

In the cross‐sectional study, we conducted an online satisfaction survey for this group of course participants to obtain their feedback as subject matter experts. Aiken's content validity coefficient (CVC) was calculated to assess the content validity. Cronbach's *α* coefficient was determined to explore the survey's reliability. IBM®‐SPSS® version 26 was utilized to explore the data.

**Results:**

The SEM satisfaction survey demonstrated important satisfaction with the implemented training with its robust reliability and content validity (Cronbach's *α* = 0.922 and CVC = 0.952). The participants also recommended additional related topics.

**Conclusion:**

Sustaining and reinforcing the HazMat‐CBRN Incident Management course was strongly recommended, considering the increase of HazMat‐CBRN threats worldwide.

## 1 INTRODUCTION

Hazardous Material (HazMat) and Chemical, Biological, Radiological, and Nuclear (CBRN) incidents are environmentally precarious and perilous to living beings. They can be deliberate or accidental or follow the re‐emergence of highly contagious diseases such as the Ebola Virus Disease in West Africa in 2016 or, more recently, the novel Corona Virus (COVID‐19).^1^ The recent ammonium nitrate explosion in Lebanon in August 2020, which killed 204, injured over 6000 people and caused extensive damage leaving over 300,000 people homeless, exposed a lack of understanding of the risks associated with the incorrect storage of a volatile substance within a nearby residential area.^2^ Over the last two decades, we have had enough experience to realize that terrorist attacks cross borders and are not limited to specific countries. Furthermore, the danger associated with their creative techniques, such as chemical and biological attacks, increases in likelihood as time passes.^3^, ^4^ Undeniably, viruses can be weaponized and utilized in terrorist attacks, as was the case with Anthrax in the United States of America in 2001 and later.^5^

The State of Qatar is one of the leading players in liquid natural gas production and investment worldwide. It has a sizeable industrial activity network^6^ with four major industrial cities specializing in refining and liquefaction of gas, chemical plants, and manufacturing goods that require gas as a feedstock or for energy: Dukhan Petroleum City, Ras Laffan Industrial City, Mesaieed industrial city, and Doha Industrial Area. Furthermore, Qatar has more than 116 active petrol stations (fixed and mobile) as of 2021, including three marine petrol stations,^7^ all distributed across the 11,571 km^2^ surface area and coastline of the country. This may raise the risk of an “Explosive Atmosphere” (accumulation of flammable gas, mist, dust, or vapors combined with air).^8^

Hamad Medical Corporation Ambulance Service (HMCAS) is a governmental organization delivering pre‐hospital healthcare in the State of Qatar. In emergencies, HMCAS Emergency Medical Dispatchers (EMD) operating from the National Command Center (NCC) receive the emergency call then, upon identifying the caller's address, they immediately dispatch the nearest medical emergency response unit (ERU), collect further information from the caller and identify accordingly the additional appropriate resources required (Critical Care Paramedic unit, Ambulance Service helicopter team, Civil Defense).^9^ After that, EMDs assist the caller in helping the victim by providing the pre‐arrival instructions until the arrival of the dispatched ERU. Some ERUs are manned by two ambulance paramedics (APs), ERU with critical care assistant (CCA) and critical care paramedic (CCP), and others with a distribution supervisor (credentialed at AP level).^9^, ^10^ They are dispatched according to the case coding determined initially by the EMDs inputting data into a computerized algorithm.^11^ In addition, HMCAS Specialized Emergency Management (SEM) section personnel can be dispatched in a major incident. They are experienced APs trained in pre‐hospital disaster management, including HazMat‐CBRN emergencies. They regularly attend Major Incident Medical Management and Support (MIMMS) courses, Hazardous Material First Responder (Awareness and Operation level) training from the Texas A&M Engineering Extension Service (TEEX®), decontamination training from Decontamination Education and Consulting on Nuc/Bio/Chem (DECON LCC®).^12^, ^13^, ^14^ In the case of a HazMat‐CBRN incident in the State of Qatar, HMCAS personnel will be the primary responding healthcare force.

Many recent studies emphasized the importance of having well‐trained and prepared healthcare workers (HCWs) when responding to HazMat‐CBRN incidents.^15^ Hence, HMCAS has established a “Hazardous Material Incident Management” course accredited by Qatar's Ministry of Public Health (MoPH) Department of Healthcare Practitioners (DHP). The course was created to be delivered to all HMCAS HCWs as first responders and also incorporated HCWs from private hospitals in Qatar.^9^, ^16^

The study aims to explore the reliability and validity of the satisfaction survey answered by HMCAS SEM personnel as subject matter experts about the “HazMat Incident Management” course and seek their opinion regarding the HMCAS HCWs' needs regarding other HazMat‐CBRN‐related topics.

## 2 MATERIALS AND METHODS

### 2.1 Study design

This cross‐sectional study used a reliable and validated satisfaction survey for the HMCAS SEM personnel who participated in the “HazMat Incident Management” course. The HMC Medical Research Committee approved this study under the reference number MRC‐01‐20‐372. Participation in the satisfaction survey was considered consent to collate responses for anonymized and aggregated analysis.

#### 2.1.1 Prestudy procedure

The knowledge and proficiency of pre‐hospital HCW in managing HazMat‐CBRN incidents seem crucial in determining the safety and efficiency of on‐scene responders within the first minutes of an incident.^17^ Therefore, understanding the extent of HCW's knowledge about a HazMat scene with the other risk factors when dealing with HazMat‐CBRN incidents is essential for a safe and successful response.

**Figure 1:**
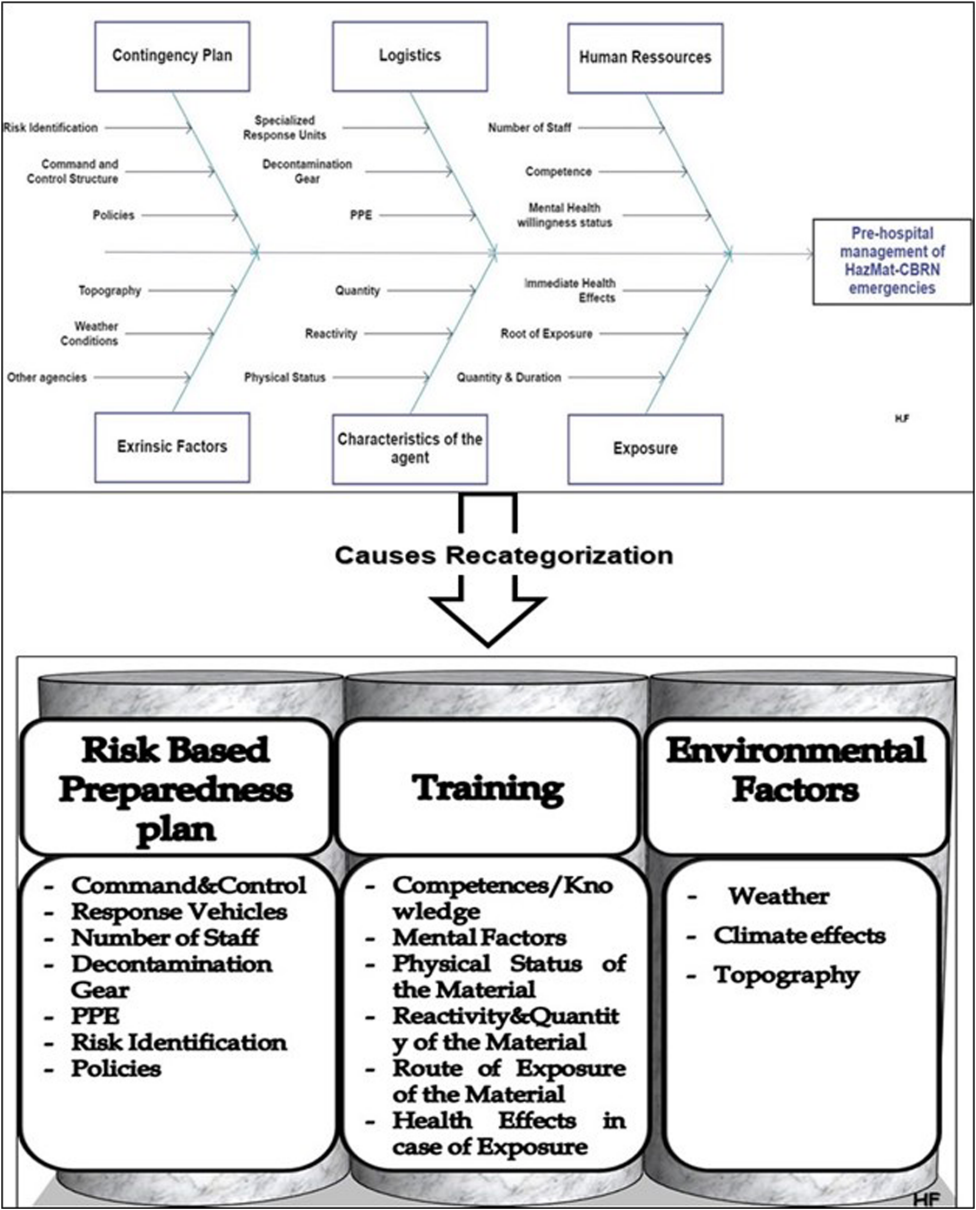
Ishikawa diagram and causes recategorization

Therefore, first, the Ishikawa diagram (Figure 1) was used in this study to categorize the factors and risks that can affect the pre‐hospital system's readiness to manage HazMat‐CBRN incidents. It is also known as the “cause‐and‐effect diagram” or “Fishbone diagram.” It has been considered an essential tool in identifying a problem's root causes and understanding the correlation between their contributing factors.^18^ It is used frequently in the quality improvement and risk assessment domains. It helps recognize potential causes of process variation (environment, logistics, human resources, system) and determine corrective actions. In our studies, it helped to identify the fundamentals of pre‐hospital response readiness for potential HazMat‐CBRN incidents

The causes identified in Figure 1 were categorized into the three main pillars: risk‐based preparedness plan, training, and environmental factors.

Second, the three pillars of successful management of HazMat‐CBRN incidents in pre‐hospital settings were categorized as extrinsic and intrinsic factors.

*Extrinsic factors*, mainly environmental factors. They are out of the control of HMCAS and hence can only be identified and monitored^19^, ^20^ (Figure 1).

*Intrinsic factors* are factors that HMCAS can control. They include a risk‐based preparedness plan and training.

First, *risk‐based preparedness* is an essential part of a successful contingency plan for HazMat‐CBRN incidents to mitigate the risks of emergency response and reduce the odds of HCWs exposure to HazMat‐CBRN agents. This can be achieved by continuous training and simulation.^21^ Some factors related to a risk‐based preparedness plan can also be considered extrinsic (other agencies, liquefied natural gas facilities…).

Second, for *training*, researchers emphasized that pre‐hospital HCWs must acquire sufficient knowledge and skills to effectively manage HazMat‐CBRN incidents.^22^ Therefore, the “HazMat Incident management” course was designed and delivered to fulfill the HMCAS personnel's needs.^9^ The course material was developed based on input from the HMCAS SEM and other pre‐hospital healthcare personnel. The objectives were to:

− Introduce personnel to the HMCAS HazMat‐CBRN incident management strategy.
− Educate personnel, including those working in NCC, to rapidly identify CBRN agents so they can deploy and use the appropriate resources.
− According to its severity level, introduce the resources available at HMCAS to manage a potential HazMat‐CBRN incident.
− Familiarize HMCAS personnel with the Emergency Response Guidebook (ERG) to establish primary safety measures and build their response plan.^23^
− Improve the coordination between HMCAS EMDs and pre‐hospital responders (AP, CCA, CCP, and distribution supervisors) by participating in the role‐play and interactive case discussion sessions.

The “HazMat Incident Management” course also included pre‐ and postcourse multiple‐choice assessment tests to evaluate this course's impact on improving the participants' knowledge. It was a one‐day course delivered on 19 occasions from June 2019 to June 2020 with a maximum group size of 15–20 participants to ensure the delivery of a quality program. About 262 HMCAS personnel registered for the course, but only 72.14% (*N* = 195) were able to attend. 23.07% (*N* = 45) were HMCAS SEM personnel.^9^

**Figure 2:**
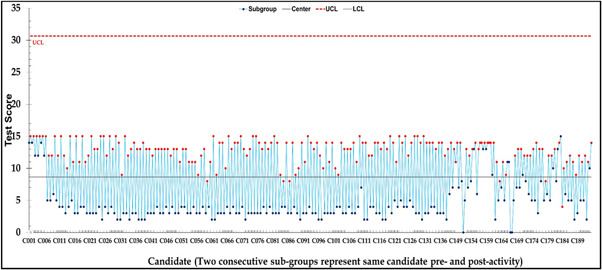
Candidate paired pre‐ and postcourse tests scores

The Shewhart Statistical Process Control chart is shown in Figure 2 (Prepared using Microsoft Excel®), demonstrating that this course's impact consistently improved the participants' knowledge about pre‐hospital management of HazMat‐CBRN incidents. In Figure 2, the precourse test scores were marked in blue. The postcourse tests score were marked in red. Some participants could not attend the pre‐ or postcourse assessment; thus, there are missing values.

#### 2.1.2 Intervention

A satisfaction survey was circulated to understand the fulfillment of the “HazMat Incident Management” course's intended objectives and whether it should be sustained and generalized to all HMC HCWs involved in pre‐and in‐hospital management of similar incidents. It was conducted to explore participants' opinions about the course.

First, a qualitative and quantitative review of the survey content was conducted by a panel of seven experts (Table 1) in relevant fields (three in research and education, two HazMat‐CBRN incidents response, and two in disaster management).

**Table 1:** Summary of content validity determination steps

1. Expert panel members' background			
**Experts**	**Background**	**Experts**	**Background**
**Expert1**	Director of Research at HMCAS Professor of Simulation in Healthcare Education at the University of Hertfordshire (UK) PhD in simulation in healthcare education. Master's degree in Applied Physics Post‐Graduate Certificate in Teaching and Learning in Higher Education	**Expert5**	− Head of Profession in HMCAS − PhD, which looked at the management of patients following exposure to a CBRN agent
**Expert2**	Medical Doctor. Group Head of Clinical Governance, Risk Management, and Quality Improvement Senior Consultant in HMCAS Improvement Advisor	**Expert6**	− PhD in Emergency Medicine − Master's degree in Business Administration (MBA) − Senior consultant in HMCAS
**Expert3**	Ambulance Paramedic Senior operations manager IN hmcas Instructor in Major Incident Medical Management and Support (MIMMS)	**Expert7**	− Instructor MIMMS − Master's degree in Executive Business Administration − Communication Manager in HMCAS
**Expert4**	Master's degree in Emergency and Disaster Management Manager in HMCAS		

2. **HMCAS experts' panel rating**										
	**Q1**	**Q2**	**Q3**	**Q4**	**Q5**	**Q6**	**Q7**	**Q8**	**Q9**	**Q10**
**EXP1**	5	5	5	5	5	5	5	5	5	5
**EXP2**	5	5	5	5	5	5	5	5	5	5
**EXP3**	5	5	5	5	5	5	5	5	5	5
**EXP4**	5	5	3	5	5	5	5	5	5	5
**EXP5**	5	5	5	5	5	3	4	4	4	4
**EXP6**	4	4	4	4	5	5	5	5	5	5
**EXP7**	5	4	4	5	5	5	4	4	5	5
	**Q11**	**Q12**	**Q13**	**Q14**	**Q15**	**Q16**	**Q17**	**Q18**	**Q19**	**Q20**
**EXP1**	5	5	5	5	5	5	5	5	5	Suggested and agreed by the panel.
**EXP2**	5	5	5	5	5	5	5	5	5	
**EXP3**	5	5	5	5	5	5	5	5	5	
**EXP4**	5	5	5	5	5	5	5	5	5	
**EXP5**	4	5	5	4	4	4	4	5	5	
**EXP6**	5	5	5	5	5	5	5	5	5	
**EXP7**	5	5	3	5	5	5	4	5	5	

3. **Aiken V content validity coefficient (cvc) values**									
**2.1. Items Aiken V CVC**					**2.2. Confidence interval of items CVC score**				
**Item**	**CVC**	**Validity**	**Item**	**CVC**	**Validity**			**Statistic**	**Standard error**
**Q1**	0.964	Valid	**Q11**	0.964	Valid	Mean		0.95489	0.008094
**Q2**	0.929	Valid	**Q12**	1	Valid	95% Confidence interval for mean	Lower Bound	0.93789	‐
**Q3**	0.857	Valid	**Q13**	0.929	Valid		Upper Bound	0.9719	‐
**Q4**	0.964	Valid	**Q14**	0.964	Valid	5% Trimmed Mean		0.95783	‐
**Q5**	1	Valid	**Q15**	0.964	Valid	Median		0.964	‐
**Q6**	0.929	Valid	**Q16**	0.964	Valid	Minimum		0.857	‐
**Q7**	0.929	Valid	**Q17**	0.929	Valid	Maximum		1	‐
**Q8**	0.929	Valid	**Q18**	1	Valid	Range		0.143	‐
**Q9**	0.964	Valid	**Q19**	1	Valid	Skewness		−0.946	‐0.524
**Q10**	0.964	Valid				Kurtosis		1.993	1.014

*Note*: Aiken V CVC conclusion: Mean Aiken V CVC = 0.952; Another question (Q20), suggested by the experts, was added.

Due to the COVID‐19 pandemic restrictions, an invitation letter attached with an evaluation sheet in a fillable PDF form was sent by e‐mail to the experts. They were invited to evaluate the relevance of each item by rating them using a 5‐point Likert scale (1 = Not Relevant, 2 = Moderately Relevant, 3 = Undecided, 4 = Relevant, 5 = Strongly Relevant) and return it by e‐mail. They were also asked to provide suggestions for each item on the same form.

Second, this satisfaction survey hosted on a Google Form was sent as a phone message hyperlink to all participants in July 2020.

The survey included five demographic questions about the participants' gender, age, job title, previous courses they received in the disaster management/HazMat‐CBRN field, and experience at HMCAS. It also included 12 questions on the participants' opinions about the course.^24^ They were based on a 5‐point Likert scale, so it was easy to answer and allowed participants to rate several aspects of the course.^24^ Furthermore, the survey included another Likert‐scale question asking the participants if they would recommend the training course to all HMC personnel. The participants were also asked about any other specific training themes in HazMat‐CBRN health emergencies they would recommend receiving in future training. The consent form was sent to the participants via e‐mail, and they were asked to reply to the same e‐mail with their participation approval or rejection within 3 days.

### 2.2 Participants and sampling

The survey targeted the HMCAS SEM personnel in particular as they are specialized and well‐trained in dealing with mass casualty incidents, including HazMat‐CBRN emergencies and participated in the HazMat Incident Management course.

At the time of this study, around half of this team were pre‐hospital HazMat‐CBRN first responders. The remaining personnel worked closely with HazMat‐CBRN first responders in a support capacity. They are routinely retrained and retested through monthly practical exercises.

Purposive sampling was used for this study. Hence, only HMCAS SEM section personnel with the following criteria were included:

− Participants of the “HazMat Incident Management” course.
− In addition, they undertook at least one HMCAS‐supported pre‐hospital HazMat‐CBRN/disaster preparedness and response training before completing the “HazMat Incident Management” course. The HMCAS‐supported training included:

- The HazMat First Responder training offered by Texas A&M Engineering Extension Service.
- The CBRN decontamination training offered by Decon‐LLC consulting services.
- The Advanced HazMat Life Support Course (AHLS).
- The 1‐day basic training organized previously by HMCAS about donning and doffing in CBRN incidents.

The HMCAS SEM personnel who did not participate in the “HazM Incident Management” CPD course were excluded from this study.

### 2.3 Statistical methods

First, certain statistical indexes were measured to explore the internal and external consistency of the survey's items (The survey's content validity and reliability).

C*ontent validity* refers to the degree to which the items in a survey represent the subject being assessed.^25^ Based on the experts' scoring, content validity was verified by calculating the *Content Validity Coefficient (CVC)* using the ***Aiken V formula*** below.^26^

$$“\mathrm{Aiken}\;V\;\mathrm{formula}”:V=\sum \frac{\left(R-\mathrm{Lo}\right)}{E\left(\mathrm{Hi}-1\right)},$$

where R is the rate value given by the expert; Lo is the Lowest Validity Score value; E is the number of experts involved; Hi is the Highest Validity Score Value.

Aiken V value ranges between 0 and 1. An item is declared valid if this coefficient is at least equal to 0.6. An item with an Aiken V coefficient less than 0.6 is not considered valid.^27^

*The reliability test* is a statistical tool to measure the internal consistency of the survey's items (whether the survey remains consistent over repeated trials of the same subjects under identical conditions).^28^ The reliability coefficient ranges from 0 (No reliability) to 1 (Excellent reliability). It is measured by determining Cronbach's *α* coefficient for a Likert‐scale survey.^29^ The Cronbach's *α* is judged as unsatisfactory (Cronbach's *α* ˂ 0.5), poor (0.5 ≤ Cronbach's *α* ˂ 0.6), acceptable (0.6 ≤ Cronbach's *α* ˂ 0.7), good (0.7 ≤ Cronbach's *α* ˂ 0.9), and excellent (Cronbach's *α* ≥ 0.9).^28^, ^30^

When calculating Cronbach's *α*, the interitem correlation is also determined, which helps to estimate the level of correlation between all pairs of items, then assessing the level of interitem homogeneity as it affects the value of Cronbach's *α*. The optimal range of average interitem correlation is 0.15–0.50. If the then interitem correlation is less than 0.15, then the items are with bad correlation. If above 0.5, the items are repetitive.^25^, ^29^

Second, Descriptive statistics were conducted to explore the outcome.

The data were explored using IBM®‐SPSS® version 26.

## 3 RESULTS

Forty‐five personnel from the HMCAS SEM section met the inclusion criteria. 100% (*n* = 45) of the targeted population attended the course and answered the survey.

### 3.1 Exploring the content validity

The content validity in this study was determined using quantitative analysis of expert judgments and qualitative expert reviews (Table 1). It was suggested to add another question asking the SEM personnel if they believed that the rest of the HMC personnel should attend this course periodically.

Based on the experts' rating in Table 1, Aiken V CVC for content validation was calculated and was shown in the same table.

Using the Aiken V formula, CVC was calculated. Based on the total number of experts and Table 1, the content of this survey's items was strongly valid as the mean of *CVC = 0.952* with a narrow *confidence interval of 0.937–0.971*.

### 3.2 Exploring reliability

The reliability was assessed by calculating the Cronbach α coefficient for the Likert‐scale survey. As per Table 2, Cronbach's *α* = 0.883 with a robust interitems correlation.

**Table 2:** Summary of reliability test

1. Reliability statistics			
Cronbach's *α*		Cronbach's *α* based on standardized items	*N* of items
(a)	0.883	0.901	13
(b)^a^	0.922	0.924	12

2. **Interitems correlation**													
	**Q6**	**Q7**	**Q8**	**Q9**	**Q10**	**Q11**	**Q12**	**Q13**	**Q14**	**Q15**	**Q16**	**Q17**	**Q19**
**Q6**	1	0.64	0.652	0.596	0.441	0.48	0.452	0.341	0.3	0.406	0.327	0.476	−0.098
**Q7**	0.64	1	0.874	0.778	0.592	0.432	0.38	0.169	0.35	0.512	0.472	0.296	−0.046
**Q8**	0.652	0.874	1	0.734	0.658	0.487	0.439	0.165	0.371	0.566	0.496	0.366	−0.147
**Q9**	0.596	0.778	0.734	1	0.685	0.642	0.518	0.323	0.635	0.648	0.691	0.349	−0.08
**Q10**	0.441	0.592	0.658	0.685	1	0.309	0.335	0.096	0.423	0.5	0.46	0.152	−0.162
**Q11**	0.48	0.432	0.487	0.642	0.309	1	0.592	0.56	0.492	0.553	0.536	0.676	−0.146
**Q12**	0.452	0.38	0.439	0.518	0.335	0.592	1	0.422	0.618	0.778	0.673	0.553	−0.159
**Q13**	0.341	0.169	0.165	0.323	0.096	0.56	0.422	1	0.557	0.388	0.606	0.587	0.131
**Q14**	0.3	0.35	0.371	0.635	0.423	0.492	0.618	0.557	1	0.734	0.919	0.388	−0.056
**Q15**	0.406	0.512	0.566	0.648	0.5	0.553	0.778	0.388	0.734	1	0.799	0.424	−0.192
**Q16**	0.327	0.472	0.496	0.691	0.46	0.536	0.673	0.606	0.919	0.799	1	0.422	−0.061
**Q17**	0.476	0.296	0.366	0.349	0.152	0.676	0.553	0.587	0.388	0.424	0.422	1	−0.214
**Q19**	−0.098	−0.046	−0.147	−0.08	−0.162	−0.146	−0.159	0.131	−0.056	−0.192	−0.061	−0.214	1

^a^ Cronbach's *α* when deleting Q19 due to the correlation with the rest of the items.

All items have a positive and good interitems correlation, except for Q19, which negatively correlated with all other items. When Q19 was excluded from testing its impact, Cronbach's *α* value increased from 0.883 to 0.922. The survey was strongly reliable.

### 3.3 Exploring SEM personnel responses to the satisfaction survey

The demographic data demonstrated a higher representation of males *(n=40)* compared to females (*n* = 5) gender, and they reported being well trained in the disaster management domain (including HazMat‐CBRN incidents). Before this training, 44.44% (*n* = 32) received CBRN decontamination training from DECON LLC®, 17.57% (*n* = 13) received CBRN First Responder training from TEEX®, 0.41% (*n* = 4) participated in Advanced HazMat Life Support (AHLS) course which is an international, well‐known course.^31^ In addition, 35.14% (*n* = 26) had already received Major Incident Response (MIR) Overview training, organized by HMCAS, 16.22% (*n* = 12) participated voluntarily in other CBRN courses organized outside of HMCAS, 10.81% (*n* = 8) participated in MIMMS training. The remainder received other HazMat‐CBRN‐related training or did not receive any other training.

**Figure 3:**
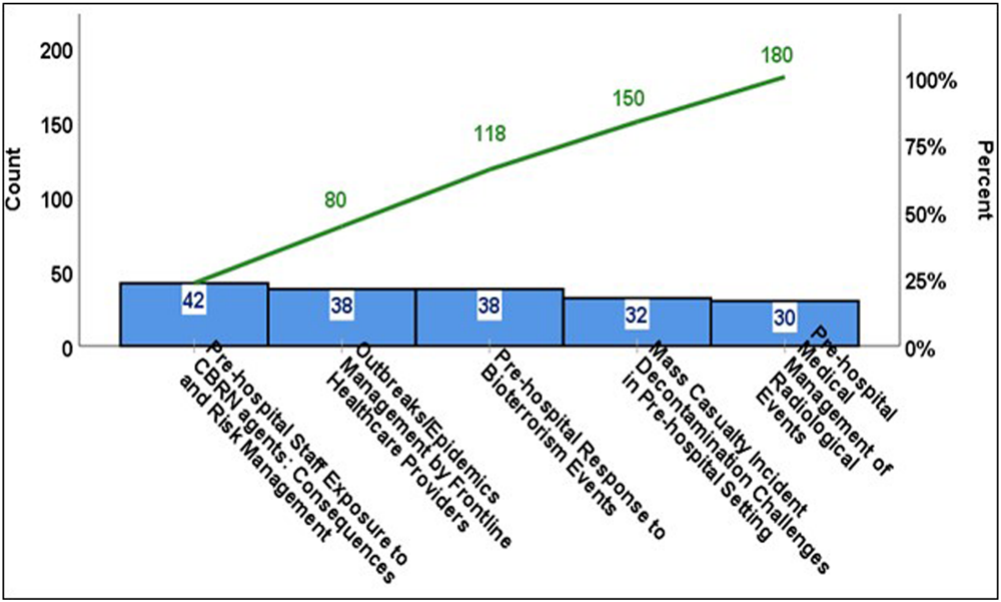
Pareto chart: Suggested future training topic by SEM personnel. SEM, Specialized Emergency Management.

91.11% (*n* = 41) indicated that they were satisfied with the time allocated for this course (6–7 h), and 100% (*n* = 45) agreed that the slides and videos created for this course were well designed. In addition, 100% (*n* = 45) agreed that the case scenarios and role‐play were well developed. They all agreed that these materials helped them understand the risks of exposure to HazMat‐CBRN agents. 91.12% (*n* = 41) were very satisfied with the information provided. Furthermore, they all agreed that this course significantly impacted their knowledge about HazMat‐CBRN incidents management and enhanced their readiness and safety to respond to such incidents. 53.33% (*n* = 24) suggested that this course should be mandated every year, whereas 42.22% (*n* = 19) suggested that it should be delivered every 2 years, and the remainder proposed (4.45% (*n* = 2) every 5 years). Participants showed their motivation and reported needing training in other topics related to HazMat‐CBRN incidents pre‐hospital management. The topics suggested were (Figure 3):

- *Outbreaks/Epidemics Management by Frontline HCWs;*
- *Pre‐hospital Medical Management of Radiological Emergencies;*
- *Pre‐hospital Personnel Exposure to CBRN agents: Consequences and Risk Management;*
- *Pre‐hospital Response to Bioterrorism Emergencies;*
- *Mass Casualty Incident Decontamination Challenges in the pre‐hospital setting*.

## 4 DISCUSSION

Pre‐hospital HCWs are involved in managing critically ill patients in the out‐of‐hospital environment; therefore, health sciences researchers emphasize the importance of equipping them with the most appropriate level of education.^32^ Heraclitus, the Greek philosopher, believes that “Change is the only constant,” and the wind of change went through educational approaches and systems. Moreover, a radical change was observed; teaching evolved from the didactic passive method to actively involving learners through workshops and case scenario discussions and recently using the e‐learning methods.^32^, ^33^, ^34^ Nevertheless, previous studies pointed out that the success of the health science education methods was based on the instructors' views.^35^, ^36^, ^37^ They considered that the instructors' experience as clinicians plays a vital role in building the educational bridge between practice and knowledge. Indeed the instructor's expertise in their field helps to repurpose the educational methods according to the observed needs. Thus in this study, we could also demonstrate that participants' expertise is crucial in deciding to review or sustain these educational methods.

In health research, it is recommended to examine a questionnaire or survey's validity and reliability.^29^ Content validity represents the integrity and accuracy of survey items covering the subject we are measuring, whereas reliability measures the firmness and the internal consistency of survey items.^29^ It helps us determine if we will have similar results if repeated.^38^

In this study, the CVC was calculated using the Aiken V formula.^26^ Based on the total number of multidisciplinary experts (7), a minimum CVC of 0.6 was required to validate an item.^28^ The benefit of this number of multidisciplinary experts' participation helps reduce the bias in evaluation.^39^ Table 1 shows the mean of *CVC = 0.952* with a strong *confidence interval of 0.937–0.971*. Consequently, the content of this survey's items is strongly valid.

A reliability test was conducted by calculating the Cronbach α coefficient for 13 Likert scale questions. As per Table 2, *Cronbach's α = 0.883* with strong interitems correlation except for item 19. This survey, in conclusion, is also strongly reliable, and we are more likely to have the same results if repeated many times.

In addition, the satisfaction survey responses showed the participants' strong interest in the HazMat Incident Management course. Based on the survey results, the implemented training met the responders' expectations.

Furthermore, considering other research studies' recommendations,^40^, ^41^, ^42^ the HazMat Incident Management course should be provided to a broader audience by including other governmental and nongovernmental health partners in Qatar, such as frontline staff from the Ministry of Interior, the Ministry of Defense.

This was also recently recommended by a Middle Eastern researcher.^43^ Pre‐hospital readiness for HazMat‐CBRN incidents demands well‐trained, skillful HCWs, and a willingness to cooperate with other medical and nonmedical responding agencies.^44^ To empower HCWs' capabilities to respond to HazMat‐CBRN incidents, they require frequent training, as experience alone may not be sufficient, and lapses.^45^

From this study, and as advised by international guidelines,^46^ with increasing worldwide risks of HazMat‐CBRN and bioterrorism threats around the world in general and in the Middle‐East‐North‐African (MENA) region in particular,^47^ we recommend not only to sustain and mandate this *“*HazMat Incident Management*”* course for all HMCAS personnel, but also to upgrade it into a more comprehensive package by adding more related topics. We also believe that expanding the audience beyond HMCAS personnel and including other personnel from the hospital, such as emergency department personnel and from the other governmental agencies, is needed as HazMat‐CBRN incidents require a multidisciplinary response, as proven by many authorities.^48^, ^49^

## 5 LIMITATION

As many important mass gathering events will be held in the Middle East and Qatar within the next few years,^50^ including the FIFA 2022 World Cup, HMCAS will continue to increase its HCW workforce capacity. This creates dynamism within the team's structures, including the HazMat‐CBRN team. Therefore, ensuring that newly recruited personnel are being trained is critical. In addition, having such activity reaccredited by DHP requires a further long administrative process. Furthermore, the COVID‐19 pandemic has affected the delivery of training courses. Delivering this activity through online channels was not an option given the resource demands in responding to the pandemic and the need for hands‐on training and skills acquisition. Many sessions have had to be canceled; hence the course could not recruit as many participants as originally intended.

Additionally, some of the instructors had resigned and moved out of Qatar. Therefore, assessing the instructors' opinions about the different components of the implemented course was not applicable. It could help us determine their satisfaction level and suggestions to improve the implemented course and, consequently, help build a more robust statistical analysis and study the differences between groups (instructors vs. participants).

## 6 CONCLUSION

At the time of this study, HMCAS succeeded in empowering pre‐hospital HCWs with the appropriate knowledge to manage potential HazMat‐CBRN incidents in Qatar, as testified by their feedback regarding the course. Sustaining and reinforcing this course is recommended. With the increasing risks of HazMat‐CBRN incidents, continuously improving the created training packages are needed to cope with the HCWs' requirements. Therefore, assessing trained participants' opinions through a valid and reliable tool can help ensure the continuous improvement of these packages.

## AUTHOR CONTRIBUTIONS

All authors have read and approved the final version of the manuscript. Hassan Farhat and Hamad Medical Corporation had full access to this study's data and take complete responsibility for the data's integrity and the data analysis's accuracy.

## CONFLICT OF INTEREST

The authors declare no conflict of interest.

## ETHICS STATEMENT

The Hamad Medical Corporation‐Medical Research Committee approved this study as part of a Quality Improvement/Audit Project (Ref: MRC‐01‐20‐372) on June 30th, 2020. Participation in the satisfaction survey was considered consent to collate responses for anonymized and aggregated analysis.

## TRANSPARENCY STATEMENT

The manuscript is an honest, accurate, and transparent account of the study being reported, that no important aspects of the study have been omitted, and that any discrepancies from the study as planned (and, if relevant, registered) have been explained.

## Data Availability

The primary author holds the anonymous data supporting this study's findings and is available for review upon request.
